# Exploring Care and Recovery for Individuals With Post-traumatic Stress Disorder: A Scoping Review

**DOI:** 10.7759/cureus.53741

**Published:** 2024-02-06

**Authors:** Jennifer R Smith, Kyle J Drouillard, Angel M Foster

**Affiliations:** 1 Faculty of Health Sciences, University of Ottawa, Ottawa, CAN

**Keywords:** care for ptsd, scoping review, recovery, qualitative research, ptsd, post-traumatic stress disorder

## Abstract

Most people experience trauma at some point in their lives. The sources of trauma can include accidents, natural disasters, physical or sexual assault, combat, torture, or the death of a loved one. Experiencing or witnessing any of these, or other terrifying events, may make one susceptible to developing post-traumatic stress disorder (PTSD), a trauma- and stressor-related mental health condition. The common symptoms and consequences of PTSD include intrusive and distressing thoughts, memories, or flashbacks related to the traumatic event; avoidance of situations, people, or activities that remind one of the traumatic event; irritability, sleep difficulties, or hypervigilance; feelings of guilt, shame, or fear; substance use; strains on relationships; and suicidal thoughts and behaviors. These consequences can have devastating effects on the individual and their family members, friends, co-workers, peers, and communities. Effectively treating PTSD, therefore, is critical not only for the individual but also for the well-being of families, communities, and society at large. However, while treatments for PTSD exist, effectively treating patients with PTSD remains elusive. Further, despite the recognition that people’s experiences are essential in understanding PTSD and provide valuable insights into what interventions are effective and how they impact recovery, patient perspectives and experiences of care and recovery have not been well-explored.

We conducted a scoping review to address the following question: what is known about the experiences and perspectives of care and recovery for individuals with PTSD? We searched the Medical Literature Analysis and Retrieval System Online (MEDLINE), Embase, American Psychological Association’s (APA) PsycInfo, the Cumulative Index to Nursing and Allied Health Literature (CINAHL), PTSDPubs, and Google Scholar for peer-reviewed and grey literature that used qualitative methods to report on the recovery or care experiences of adults with lived experiences of PTSD. We extracted information about study objectives, study characteristics, and key findings; reported summary statistics; and performed content and thematic analyses.

We identified 14 relevant studies that provide insight into the participants’ lived experiences and perspectives of PTSD care and recovery. Though limited, the body of literature sheds light on critical themes and processes in the journey of care of PTSD, which we organized into four overarching categories: pre-treatment understanding and experiences of PTSD, the experience of care or treatment, the importance of relationships and social support, and expanding the understandings of recovery.

Living with and healing from PTSD are a unique and individualized human experience of developing and redeveloping relationships with oneself, with others, and with society. The recommendations for practice include educating and establishing well-informed support networks for individuals with PTSD, training healthcare practitioners in all aspects of formal and informal PTSD treatment and care needs, ensuring a continuum of care, and understanding the human experience of PTSD.

## Introduction and background

Trauma is now recognized as a common human experience [1]; approximately three in four Canadians report experiencing at least one traumatic event in their lifetime [2]. Experiencing or witnessing a life-threatening event such as physical or sexual assault; military combat; natural disasters; terrorism; serious threats or harm to one’s children, spouse, or other close relatives or friends; sudden destruction of one’s home or property; or seeing another person being seriously injured or killed due to an accident or physical violence [3] may make one susceptible to developing post-traumatic stress disorder (PTSD [4]), a trauma- and stressor-related mental health condition [5].

PTSD has physical, psychological, social, and/or interpersonal impacts on individuals [6], families, and personal and professional communities due to higher rates of unhealthy behaviors, physical health problems, mortality, lost work and productivity, impaired relationships, domestic violence, and family strain [7,8]. Given its wide-ranging impacts, effectively treating PTSD is critically important. While many psychotherapies for PTSD have been developed over the past two decades [9-11], effectively treating patients with PTSD remains elusive [12]. Despite the recognition that patients’ insight and input in research and practice are key to responding to the needs of people living with PTSD [13], patient perspectives on the journey to recovery and ameliorating PTSD symptoms have not been well-explored.

Therefore, the purpose of this scoping review is to synthesize the available qualitative research on the experiences and/or perspectives of individuals living with PTSD. We aim to explore what has been most helpful in their recovery journey, determine the overall state of research activity in this area, and identify gaps and future directions for research and practice. This review is directed by the following research question: what is known about the experiences and perspectives of care and recovery for individuals with PTSD?

## Review

Methods

Our scoping review protocol follows the framework initially described by Arksey and O’Malley [14], which was later refined by Levac et al. [15]. As such, we defined the research question according to the study purpose and desired outcomes; identified and selected relevant studies using an iterative approach; charted data by incorporating numerical summaries and qualitative thematic analysis; and collated, summarized, and reported on the results, reflecting on the implications for research and practice. We enlisted a digital literacy librarian with expertise in designing exhaustive database searches and followed the Preferred Reporting Items for Systematic Reviews and Meta-Analyses (PRISMA) guidelines for scoping reviews (PRISMA-ScR [16]) to ensure the inclusion of essential reporting items. Before proceeding with our review, we conducted an initial search to ensure that the research question was both novel and relevant.

Eligibility Criteria

We included materials in this review if they primarily used qualitative methods, including interviews, focus group discussions, and personal accounts, and reported on the recovery or care experiences of adults (i.e., >18 years of age) living with PTSD, whether clinically diagnosed or self-diagnosed. We did not limit the review to peer-reviewed journals (i.e., we did not exclude unpublished theses or books), by geographical location, or by a defined timeframe. We excluded studies exploring the experiences of adolescents and/or children (i.e., <18 years old), abstracts, and studies that interviewed the participants for the primary purpose of clinical or psychiatric assessment.

Information Sources and Search Strategy

We searched five databases relevant to our research question for peer-reviewed materials, including the Medical Literature Analysis and Retrieval System Online (MEDLINE) (Ovid), Embase (Ovid), American Psychological Association’s (APA) PsycInfo (Ovid), the Cumulative Index to Nursing and Allied Health Literature (CINAHL) via EBSCO, and the US Department of Veterans Affairs’ PTSDPubs. We performed a comprehensive three-stage search of the literature from which we identified relevant studies for our review. To begin, we conducted a limited preliminary search in MEDLINE to identify key words and subject headings pertinent to our research question. We cross-referenced our search strategy with three target articles included a priori to ensure our search was comprehensive. We subsequently translated our MEDLINE search, including subject headings, Boolean operators, and truncation, to meet the search requirements of the other four databases. Next, we searched our five chosen databases, targeting titles and abstracts of peer-reviewed materials published to the databases on or before January 9, 2023. Once we identified materials for inclusion in our review, we conducted a “hand search” of reference lists and bibliographies to identify additional materials. Finally, we searched for grey literature, including reports, essays, and scholarly research not captured in databases of peer-reviewed research. Specifically, we searched Google Scholar and websites of agencies that fund, report on, or conduct PTSD research.

We used key words, index terms, and subject headings tailored to each database to identify relevant studies. While we adapted our search terms to each database, our general strategy included nine primary terms and their variations: post-traumatic stress disorder, PTSD, qualitative, interview, narrative, focus group, psychotherapy, recovery, and process. Table 1 provides an example of one of the five search strategies performed.

**Table 1:** Example Search Strategy for Ovid MEDLINE PTSD, post-traumatic stress disorder; MEDLINE, Medical Literature Analysis and Retrieval System Online

Line number	Subject headings and key words
1	Stress Disorders, Post-Traumatic/
2	(posttrauma* stress or post-trauma* stress or PTSD).ti,ab,kf.
3	1 or 2
4	qualitative research/
5	interview/ or personal narrative/
6	focus groups/ or interviews as topic/
7	(focus group* or interview*).ti,ab,kf.
8	qualitative.ti,ab,kf.
9	personal narratives as topic/
10	narrative*.ti,ab,kf.
11	or/4-10
12	recover*.ti,ab,kf.
13	psychotherapeutic processes/
14	process*.ti,ab,kf.
15	or/12-14
16	3 and 11 and 15
17	limit 16 to "all adult (19 plus years)"

Study Selection

After importing the results from each database to Covidence (Veritas Health Innovation, Melbourne, Australia), a systematic review management tool, the principal and second reviewers (JRS and KJD) independently screened titles and abstracts to determine the broad eligibility of materials. The materials that appeared to meet all inclusion criteria, or were ambiguous or unclear to the reviewers, advanced to a full-text review. We flagged and removed any duplicates missed in Covidence’s automated search importation process. JRS and KJD held consensus meetings to resolve rare disputes regarding inclusion; AMF was available to adjudicate disputes. JRS conducted all hand searches and a thorough full-text review of consequent materials and developed and carried out a search strategy for Google Scholar using the same three-step selection process outlined previously. Consistent with the guidance for scoping reviews, we did not appraise methodological quality or the risk of bias of the included articles [17].

Data Collection, Charting, Synthesis, and Analysis

JRS reviewed and collected data from each of the included studies and used Microsoft Excel® (Microsoft® Corp., Redmond, WA) to chart pertinent data from each source. KJD conducted an independent charting of data from two of the included articles to check for consistency. JRS synthesized information about included materials, summarized participant characteristics using descriptive statistics, and conducted content and thematic analyses of the qualitative data presented in the source articles [18]. This process allowed us to summarize study populations and explore the body of literature on patient experience and/or factors influencing care or recovery for individuals with PTSD.

Results

Nature of Existing Literature

**Figure 1:**
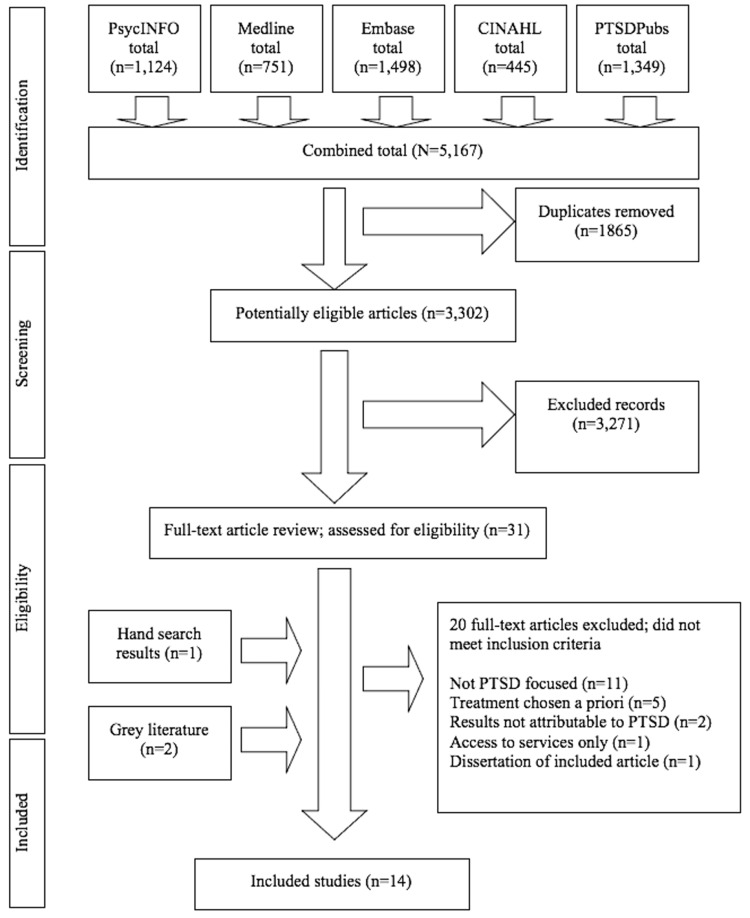
PRISMA Decision Tree PRISMA, Preferred Reporting Items for Systematic Reviews and Meta-Analyses; MEDLINE, Medical Literature Analysis and Retrieval System Online; CINAHL, Cumulative Index to Nursing and Allied Health Literature; PTSD, post-traumatic stress disorder

Our search strategy yielded a total of 5,167 potentially relevant articles from the scientific databases, of which 1,865 were duplicates and 3,290 did not meet the eligibility criteria. We eliminated most of these (n=3,271) during the first-level screening of titles and abstracts, resulting in 31 articles eligible for full-text review. Through regular consensus meetings, we identified 11 eligible articles from the database searches. JRS identified one additional study from “hand searching” reference lists and two articles through the grey literature search for a total of 34 materials eligible for full-text review. We excluded articles during full-text review because they were not sufficiently focused on PTSD (n=11), the researchers selected a therapy or treatment a priori (n=5), the participants with PTSD were not independently analyzed (n=2), the study focused solely on access to services (n=1), or the study was the dissertation of an included published article (n=1). We identified a total of 14 articles eligible for inclusion. For greater detail about our process, see the Preferred Reporting Items for Systematic Reviews and Meta-Analyses (PRISMA) flow diagram [19] presented in Figure 1.

Origin of the Evidence

In total, we selected 14 articles, published between 1993 and 2022, for inclusion in this scoping review. The studies were based primarily within the United States (n=5), the United Kingdom (n=3), and Canada (n=2), as well as one each from Croatia, the Netherlands, Portugal, and Switzerland; one article reported on multiple countries.

Study Sample and Participant Characteristics

Sample sizes ranged from one to 60, with a mean of 14 participants. The participants’ ages ranged from 19 to 72 years, and 67% of the total study population identified as men/male, and the rest were women/female. No sources reported that the participants self-identified as transgender or gender diverse. The articles did not disaggregate participant demographics by race or cultural context, outside of military culture.

Studies predominantly focused on the participants recovering from PTSD related to military service (n=7). The remaining studies focused on the participants with a range of traumatic experiences, including those directly exposed to war, those who all experienced early childhood trauma and at least two later-life traumatic events, nurses, rape survivors, and public safety personnel. There was an absence of exploratory research focused on the experiences of PTSD care or recovery in additional risk groups, such as those who have experienced domestic or intimate partner violence; survivors of unexpected events, such as car accidents, fires, or natural disasters; and populations with complex trauma.

Our review included 14 published articles, 11 of which were peer-reviewed, and three were completed in partial fulfillment of a master’s (n=2) or doctorate (n=1) degree. Eleven articles presented original research; the remaining articles were narratives (n=2) or evidence-informed commentary (n=1). The study objectives varied across articles. Six of the 14 selected articles explored concepts of recovery, e.g., how veterans view recovery [20], the experience of progressing through treatment toward recovery [21], key resources and processes to which people attributed their recovery from post-traumatic symptoms [22] or chronic PTSD [23], veterans’ perspectives on barriers and facilitators to recovery [24], and the recovery process in female veterans suffering from PTSD resulting from military/service-related sexual abuse [25]. The remaining articles centered on experiences of successfully managing PTSD and PTSD symptoms (n=2 [26,27]), complex PTSD (CPTSD) symptom management in everyday life (n=1 [28]), healing from PTSD (n=2 [29,30]), experiences with PTSD-related care (n=2 [31,32]), and lived experiences of post-traumatic growth (n=1 [33]). We provide a summary table of study and sample characteristics, as well as key findings, in Table 2.

**Table 2:** Study and Sample Characteristics (N=14)

Primary author (date)	Study aim/objective	Country of study	Sample size (n)	Percentage of males	Sample population	Data collection method/approach	Key findings
St. Cyr et al. (2022) [21]	To explore the conceptualizations of recovery. To study 1) how veterans nearing the completion of treatment for military-related post-traumatic stress disorder (PTSD) view recovery and 2) the experience of progressing through treatment toward recovery	Canada	9	100	Military	Quantitative self-report tools; semi-structured interviews	Individual reflections of “recovery” were not always aligned with the quantitative assessments of symptoms. Recovery from military-related PTSD was not viewed as a binary outcome (i.e., recovered versus not recovered); recovery was seen as a dynamic, non-linear process. Seven themes regarding recovery, organized chronologically
Parry et al. (2021) [27]	To explore veterans’ experiences of successfully managing PTSD	United Kingdom	6	83	Military	Semi-structured interviews	Managing PTSD bound up with veterans’ experiences of renegotiating their identity. The participants sought to speak about their challenges with others who understood the military context and felt that their experiences made them a valuable resource to others. Three themes developed
van der Ham et al. (2021) [31]	To explore the participants’ experiences with PTSD-related care among people with visual impairment and PTSD	Netherlands	18	22	People with visual impairment	In-depth interviews	(Mental) Healthcare providers were regularly not used to communicating with people with a visual impairment; adaptations to existing treatments needed to be suitable and accommodating to people with visual impairment
Stadtmann et al. (2018) [28]	To study symptom management in everyday life by exploring and reconstructing the views, perceptions, experiences, facilitators, and barriers of adults with complex PTSD	Switzerland	17	24	Early childhood trauma+later traumas	Semi-structured interviews	The process of symptom management was extremely exhausting for the participants; they felt very alone and were eager for support, both financial and from health professionals. Delineated the process of the sequenced progression and experiences of the participants through their life of coping with complex PTSD symptoms
Gilberto (2017)[20]	To better understand how veterans of the Canadian Forces diagnosed and treated for duty-related PTSD at the Operational Stress Injury Clinic view recovery	Canada	3	100	Military	Semi-structured interviews	Qualitative inquiry provides the rationale for changing the way in which the voice of the veteran can be heard. Some dissonance between where a person may be at in his or her recovery after trauma treatment versus where others may expect this person to be. The systems in which clients may be involved have not been structured in a way that permits the fluidity and flexibility that is the lived experience of recovery
Ferrajão and Aragão Oliveira (2016) [23]	To explore the key resources and processes attributed to recovery from chronic PTSD by a sample of veterans presenting mental sequelae of the war	Portugal	60	100	Military	Quantitative measures; in-depth semi-structured interviews	Recovered participants verbalized higher “mentalization ability” (capability for self-awareness of their own and others’ mental states). Four themes described strategies and factors related to recovery
Hatton (2016) [24]	To explore veterans’ perspectives on what they felt had aided or impeded their recovery	United Kingdom	9	100	Military	Semi-structured interviews	The concept of recovery felt inappropriate, and the participants preferred to consider their life post treatment as a continual journey of coping with their PTSD. Stigma surrounding mental health difficulties within the armed forces and the society remains. Four superordinate/master themes emerged; multiple subthemes
Palmer et al. (2016) [33]	To examine and to gain a richer understanding of the lived experience of post-traumatic growth in UK veterans who have experienced military trauma and subsequently received treatment for symptoms of PTSD	United Kingdom	8	100	Military	Quantitative measure; semi-structured interviews	Post-traumatic growth was experienced following treatment for post-traumatic stress disorder symptoms and accompanied with a commitment to change and social support. Two key themes; multiple subthemes
Vernon (2016) [32]	The author shares a personal account of his experience with PTSD and PTSD-related care so as to help others in their recovery journey	United States	1	100	Public safety personnel (firefighter/ paramedic)	Commentary (personal account)	Psychological healing takes comparatively longer than the healing of physical wounds. Seeking the right practitioner to help is as important as realizing one needs help in the first place. There is a strong desire and need for ongoing support
Ajdukovic et al. (2013) [22]	To explore key resources and processes to which people traumatized by war attribute their recovery from post-traumatic symptoms and overcoming the war-related experiences to be psychologically healthy	Croatia	43	54	People directly exposed to war	Quantitative measures; in-depth interviews	Highlight the importance of a strong orientation toward the future, a reciprocity in receiving and giving social support and involvement in meaningful activities (productive and valued individual). Eight themes identified to which the participants attributed their recovery from post-traumatic symptoms
Kuznicki (2013) [29]	The author analogizes her personal experience to a 12-step theoretical model to illustrate the reintegration process as a healing intervention for PTSD	United States	1	0	Rape survivor	Personal account; theoretically informed	If individuals cannot access their emotions in order to move through the healing process, they will remain in pain. Modelled the healing process of reintegrating split parts of oneself alongside Campbell’s 1949 12-stage model of the innate pattern of the physical, mental, and spiritual life of the human being
Bush (2010) [30]	The author shares her personal experience of PTSD and recovery as an oncology nurse who has cancer and PTSD	United States	1	0	Nurse (oncology nurse with cancer)	Commentary (lecture/literature-informed)	The experience of cancer differs from other PTSD-related trauma due to the multiple crises that occur along the cancer trajectory (not one discrete stressor). The ability to give and receive support is a key component of recovery
Wing and Oertle (1999) [25]	To determine the recovery process in female veterans suffering from PTSD resulting from military/service-related sexual abuse	United States	16	0	Military (military-related sexual abuse)	Interviews (open-ended)	The participants transformed self by progressing through five stages. In the basic social process of “transforming” or “affirming self,” the participants embraced the role of survivor and made a commitment to positive growth and change for self and others
Bille (1993) [26]	The author shares his personal story as a “hidden victim” of traumas experienced many years ago and his experience of recovering from both alcoholism and PTSD	United States	1	100	Nurse (in the Vietnam war)	Commentary (personal account)	The author uses the letters of “recovering” to summarize milestones along the road traveled toward recovery. Like the traits of PTSD, the traits of recovering often overlap and are not mutually exclusive

Key Findings

Our synthesis of the key findings, principal themes and subthemes, concepts, factors, and processes from the source materials provides insight into four overarching categories: pre-treatment understanding and experiences of PTSD, the experience of care or treatment, the importance of relationships and social support, and expanding understandings of recovery.

Pre-treatment Understanding and Experiences of PTSD

The participants often did not recognize their PTSD symptoms or attributed them to other causes [21,30-32]. Before the participants knew something was wrong, they expressed challenges related to the awareness of their PTSD [21] and exercised numerous and varied strategies to cope with current stressful events and the symptoms they were experiencing [22]. This was recognized as the “main phenomena” or the “emotional ignorance” process phase [28]. The participants reported initial denial and a lack of awareness [21,27] of a greater psychological difficulty or having no conscious memory of the basis for their discomfort [25]. The participants with CPTSD identified their symptoms as part of their personality and their being [28]. Avoidance, the denial of feelings of fear, social isolation and seclusion, and comparing their situations with others they perceived as “worse” were categorized as “emotion-focused coping strategies” [23]. The participants sought to hide their pain to avoid feeling weak [27]. Substance use, to avoid dealing with their feelings, was a common theme [21,23,25,26], and alcohol abuse, violent behavior, self-harm behavior, and suicide attempts were categorized as “acting out strategies” [23]. In Kuznicki’s 12-step process of healing from PTSD [29], modelled after Campbell’s 1949 monomyth of the innate pattern of the physical, mental, and spiritual life of the human being, The Hero’s Journey [34-36], this is the first of the 12 steps, in which the participants try to live a “normal” life [29].

When minimizing emotions or strategies to subconsciously cope, suppress, or distract themselves from their symptoms no longer worked, many experienced collapse or exhaustion [27,28], overwhelming distress [33], or a downward spiral of feeling out of control [24] and feared that they were “going absolutely insane” or that something was intrinsically wrong with them (process phase: “overcompensation” [28]), which continued until it resulted in “paroxysm,” an involuntary outburst or a sudden increase or recurrence of symptoms [28] and, ultimately, a catalyzing crisis [24] where they could no longer deny their difficulties [27]. Conflicts with family or loved ones and being unable to work left the participants “at rock bottom,” with some having considered suicide [27]. The participants reported becoming “sick and tired of being sick” [26]. Interactions with military friends with lived experiences of PTSD, the urging of partners, and noticing changes in their social networks acted as precipitating events [21] and motivations for seeking treatment [21,26,30].

Recognizing something is wrong was considered key to beginning the recovery process [20,21,24,25,32]. Receiving a PTSD diagnosis validated many participants’ experiences and offered them reassurance that they were experiencing “something normal” and that there was something “officially wrong” [24]. The participants could begin to understand their symptoms and move toward a solution, which offered hope and relief; indeed, recognizing the experience as PTSD came as a relief to many and a reassurance that they were not going mad or were not horrible people and acted as a facilitator for breaking the silence [24]. Accepting the problem, taking responsibility, and gaining control [27] are endorsed in the themes of proactively committing to change and an open mindset to seeking help, categorized as “growth development” [33]. The progression of the above themes is aligned with steps 2-5 in Kuznicki’s The Hero’s Journey. In the second step, the hero is called to take action; in the third, the call is heard and acted on only when the hero is ready, which can “take years or even a lifetime for the hero to heed the call and turn toward it” [29]; in the fourth, a person or persons become present who will provide the hero with the strength, guidance, and courage necessary to begin the quest [34]; finally, in the fifth step, the hero may choose to cross the threshold, or he may be pushed [29].

The Experience of Treatment or Care

Formal mental health treatment included psychoeducation, psychotherapy (e.g., cognitive behavioral therapy), eye movement desensitization and reprocessing (EMDR), imaginary exposure therapy, psychiatry, and medication [22,31,32]; other forms of treatment and care reported as helpful included psychomotor therapy, mindfulness, and yoga [31]. The participants experienced relief after learning more about PTSD from mental health professionals and credited psychoeducation with shifting their perspectives [21] and giving them an explanation for their experiences [27], which fostered acceptance and increased responsibility for their emotions and actions [21].

The participants’ help-seeking and treatment processes were non-linear, challenging, and lengthy [21]. Multiple studies reported a need for PTSD-related care but underscored challenges in finding mental health professionals who were a good fit for the participants’ needs [21,26,32] and sufficiently specialized to provide treatment for the given trauma or population, such as complex traumatization or the visually impaired [31], which led to delays and barriers to treatment, including accessibility, offering preferred treatments, and ineffective communication within and around treatment [28,31]. The participants reported that accessing appropriate therapies and avoiding unnecessary medical interventions were major facilitators, while being treated for another condition instead of PTSD was a major barrier [28]. The participants saw discussing problems as necessary and helpful, if they were talking to the right people [27,32]. Vernon [32] described his experience with an ill-equipped psychologist who was a hindrance to his recovery, rather than a help. Multiple sources identified ongoing “maintenance” therapy, such as periodic check-ins with a mental health clinician, and tailoring a therapeutic program to one’s own needs as key elements in remaining in a state of recovery [20,21,24,26,32].

A positive therapeutic relationship [21] established and ensured psychological safety [22], a sense of trust and security [26], and the support necessary to break out of the fear and isolation many felt when experiencing emotions, sometimes for the first time [26,29]. Therapists who provided care beyond strict professional boundaries provided reassurance to the participants and the feeling that they were being treated like a human being [20,22]. Receiving material support such as housing and social benefits, as well as resolving civil status for refugee participants, was beneficial to managing and coping with both life stressors and PTSD symptoms, as these kinds of tangible supports helped reduce life uncertainty and thereby also increased psychological safety [22].

Veterans’ takeaways from treatment included increased self-awareness and the importance of actively engaging in their treatment as integral to effectively employ a toolkit of adaptive strategies to address PTSD symptoms. The source materials identified strategies that helped the participants actively manage PTSD, including striking an adaptive balance between avoidance and engagement [21]; “problem-focused coping strategies,” such as leisure activities, work, involvement in community activities; establishing a structured daily routine; seeking social support or interaction [22,23,27]; adjusting expectations of themselves; and using self-talk as a way to ground and calm oneself during times of extreme emotion [27].

The Importance of Relationships and Social Support

Across included studies, relationships and the social support of one’s family, friends, and informal network, as well as relationships with other soldiers and relationship with self [20], were considered critical throughout recovery and key components of the recovery process. In The Hero’s Journey, this is the sixth step, in which allies offer love, acceptance, support, and knowledge [29]. Partners played an especially supportive role and were often considered a “rock” in their most difficult times. The participants withdrew from larger social networks and prioritized maintaining mutual, supportive friendships, and fulfilling their responsibility to their families helped the participants control symptoms and remain oriented toward the future [22]. Vulnerability and honest sharing of one’s secrets with another human being take a great deal of courage [26]. The participants described “seeking validation” from a listener without fear of recrimination or judgement and “sorting through confusion” with well-informed support networks [33] of friends and peers where they could share common experiences and feelings without judgement [22,25,27]. Sharing painful memories and feelings in a safe environment, such as a support group, was considered key to completing the grieving process [26] and provided the participants with opportunities to reinterpret or reappraise traumatic events [23]. The support of others who experienced, and continued to experience, many of the same feelings, flashbacks, sleep disturbances, and anger was considered one of the most valuable “tools for recovery” [26]. Veterans described support from military peers as an integral part of their treatment and recovery [20,21,24] and demonstrated an overwhelming preference for speaking to other veterans about their difficulties [27].

Ultimately, it is time for the hero to go forward alone [29]. In step 7, the hero “leaves his allies behind and heads into the abyss. He knows it will be a long journey, likely to be filled with pain, strife, and heartache. He is fearful, but he moves forward with intention and honor of his commitment to his people or to himself” [29]. The participants who were acknowledged and encouraged by others (e.g., friends, family, mentors, co-workers, and therapists) to feel their emotions, including rage and anger, emotions that were real and necessary and should not be suppressed, considered this critical to their healing from PTSD [29], as well as to learning how to honestly and openly relate to others [26]. In step 8, the hero will either die or succeed [29,34] where “succeeding” often involved changing one’s own thinking, which takes time and is often “a chain of events that result in heightened realizations” [26], a cognitive restructuring growth experience [33], that comes with increased self-acceptance [21], positive self-affirmation [26], personality hardiness [22], perceived personal resources [23], and perceived well-being [23]. In Kuznicki’s story, “the reward was release from the heaviest burden she ever had to carry” [29].

From isolation to reconnection: “Recovered” participants showed higher mentalization capability, the “capability to correlate their behaviors to emotional states and perceive the impact of their behavior on others” [23], expressed through an increased appreciation of the external world and connecting with others [33]; striving to learn, grow, and contribute to the greater good [30]; and re-evaluating one’s sense of purpose or life meaning [30,33]. The participants found community involvement healing [22,26], where working with others in recovery acted as a reminder of recovery progress [26] while acknowledging that introspection and self-reflection about feelings, needs, and wants are also necessary [26]. By the 10th stage, “the hero has suffered tremendously both physically and mentally, but he has overcome enormous challenges, and he has grown” [29].

Expanding the Understandings of Recovery

None of the articles in this review offered an a priori conceptual definition of recovery from PTSD, and only one study provided an explanation of recovery as “the ability to navigate ongoing issues of symptom management, re-engagement with meaningful roles and social networks, and a readiness for discontinuing intensive, specialized mental health treatment” [21]. Three articles categorized the participants as “recovered” versus “unrecovered” or “not recovered” from PTSD, which was appraised using quantitative validated measures: the PTSD Checklist (PCL-5 [21]), the structured mental health assessment interview (MINI [22]), and the Clinician-Administered PTSD Scale (CAPS [23]).

St. Cyr et al. [21] highlighted discordance between the participants’ quantitatively measured PTSD status and their actual experience of recovery. The participants did not view coping and recovery from PTSD as a binary outcome but as a dynamic, non-linear process; indeed, the very concept of “recovery” felt inappropriate. The participants preferred to consider their life post treatment as a journey of coping [20-22,24], small steps, and small but meaningful successes [26], within the gradual process of time [27,33]. Recovery took perseverance [27] and was reported as an enduring, evolutionary, and never-ending journey [20] of understanding one’s reactions to trauma [33] and the normalization of symptoms in everyday life [22]. The participants saw recovery as more than treatment [20], as a unique and individualized experience of developing relationships with oneself, with others, and with society.

Discussion

This scoping review sought to find and synthesize existing qualitative literature focused on the care of individuals with PTSD to understand what is known about the experiences and perspectives of people living with PTSD and their journey to recovery. Despite the dearth of information, the qualitative literature included in this review provides rich insight into the participants’ lived experiences [22,23] otherwise inaccessible to quantitative studies. Our findings suggest that understanding the process of recovery and the factors within is critical in determining when, how, who, and what best support persons experiencing PTSD.

The lived experiences of those with PTSD show that often the first step to recovery is understanding and acknowledging that there is a problem; thus, psychoeducation for those who suffer from PTSD and its symptoms appears critical to understanding their experiences and “finding solace in knowing their symptoms have an explanation and in learning that what they were experiencing, or feeling, was completely normal” [28]. Further, enabling partners, peers, family, and friends is key to help facilitate early intervention and support, avoid crisis, and potentially mitigate the devastating toll PTSD can have on both the individual and their loved ones [37].

Finding an understanding person or therapist who is collaborative and flexible has been underscored as pivotal in the recovery process. Indeed, our findings suggest that inflexible approaches to rehabilitation and recovery can be personally and professionally detrimental [20]. The lived experiences of those with PTSD illustrate the importance of training healthcare practitioners to recognize those vulnerable to the disorder and to effectively identify PTSD symptoms in order to mitigate possible long-term impacts and provide the most appropriate and helpful treatment or care options. Further, the source materials highlight the importance of creating interventions that are person-centered; consider and respond to the multidimensional nature of PTSD; provide longitudinal, continuous care (i.e., no temporal constraints on mental health service); and acknowledge recovery as a process, where the word “recovery” as a binary construct is considered inappropriate [24].

When asked about “anything” that helped, few source materials reported in any detail about formal therapeutic interventions; most participants discussed non-therapeutic factors that were helpful to PTSD symptom recovery or symptom management, such as connecting with others with common or similar trauma-related experiences [26,32,33]. Importantly, while treatment was deemed important, “by no means was it deemed the only component to healing.... Each participant experienced transformation in their journey before they had been referred for treatment, during treatment, and after treatment had concluded. Each indicated that recovery continued to this present moment” [20]. Much like receiving a PTSD diagnosis, understanding that one may live with or manage PTSD in one’s everyday life, that it is normal to not “get over it” or “bounce back” and to continue to experience symptoms of PTSD, could potentially provide sufferers a similar sense of relief. From this place of relief or “solace,” our findings suggest that their journey of recovery becomes a less painful one, one not to be measured categorically but acknowledged, understood, and accepted as a gradual process of taking action to take back parts of oneself [29] in order to live one’s life with PTSD.

Limitations

Several potential limitations of this review may include discrepancies in the literature search process, our search strategy, and our framework. We may not have captured all relevant articles; our choice of databases influenced our search strategy and consequently the publications we gleaned from our search. The terminology we utilized in the search strategy may not have been sufficiently broad to capture all published research on the research question, particularly with respect to experiences and perspectives around the journey of care or recovery from PTSD. As the principal researcher coded the majority of the included articles, her bias may be present in the selection, extraction, charting, or translation of primary data chosen from included citations as relevant for further analysis and synthesis. Finally, our exclusion criteria also limit the review, as the target population did not include individuals under the age of 18.

We tried to minimize these limitations by carrying out a focused yet comprehensive search under the guidance of a digital literacy librarian, completing a double data extraction check on two included articles to ensure accuracy, and amalgamating the most salient points from each study in the words of the participants, as per the objective of the studies. Our three-step search strategy was rigorous and thorough, and we thoughtfully recorded and justified any modifications.

## Conclusions

Few studies exist that explore the care and recovery journey of people with PTSD. The increase in research over the last five years is a promising reflection of a greater recognition of the importance of exploratory research on patient perspectives and experiences of care and recovery for PTSD. Existing literature focuses largely on the military population. While this is indeed a population at high risk for developing PTSD and deserves attention, there is a marked absence of research exploring PTSD care and recovery among other risk groups. Taken together, the included articles, theses, and commentaries contribute to a more fulsome and expanded understanding of the care and recovery journey for people with PTSD, which is essential to developing appropriate and effective interventions and providing insight into when, how, who, and what help people with PTSD in their everyday coping, managing, and healing.
